# Alcohol Use and Prefrontal Cortex Volume Trajectories in Young Adults with Mood Disorders and Associated Clinical Outcomes

**DOI:** 10.3390/bs12030057

**Published:** 2022-02-22

**Authors:** Dylan E. Kirsch, Valeria Tretyak, Vanessa Le, Ansley Huffman, Kim Fromme, Stephen M. Strakowski, Elizabeth T.C. Lippard

**Affiliations:** 1 Department of Psychiatry and Behavioral Sciences, Dell Medical School, University of Texas, Austin, TX 78712, USA; valeria.tretyak@utexas.edu (V.T.); vanessa.le@austin.utexas.edu (V.L.); akh.huffman@gmail.com (A.H.); steve.strakowski@austin.utexas.edu (S.M.S.); 2 Waggoner Center for Alcohol and Addiction Research, University of Texas, Austin, TX 78712, USA; fromme@utexas.edu; 3 Institute for Neuroscience, University of Texas, Austin, TX 78712, USA; 4 Department of Psychology, University of Texas, Austin, TX 78712, USA; 5 Institute of Early Life Adversity Research, University of Texas, Austin, TX 78712, USA

**Keywords:** bipolar disorder, depression, alcohol drinking, magnetic resonance imaging, prefrontal cortex, young adult, brain development

## Abstract

(1) Background: Alcohol use in the course of mood disorders is associated with worse clinical outcomes. The mechanisms by which alcohol use alters the course of illness are unclear but may relate to prefrontal cortical (PFC) sensitivity to alcohol. We investigated associations between alcohol use and PFC structural trajectories in young adults with a mood disorder compared to typically developing peers. (2) Methods: 41 young adults (24 with a mood disorder, age_mean_ = 21 ± 2 years) completed clinical evaluations, assessment of alcohol use, and two structural MRI scans approximately one year apart. Freesurfer was used to segment PFC regions of interest (ROIs) (anterior cingulate, orbitofrontal cortex, and frontal pole). Effects of group, alcohol use, time, and interactions among these variables on PFC ROIs at baseline and follow-up were modeled. Associations were examined between alcohol use and longitudinal changes in PFC ROIs with prospective mood. (3) Results: Greater alcohol use was prospectively associated with decreased frontal pole volume in participants with a mood disorder, but not typically developing comparison participants (time-by-group-by-alcohol interaction; *p* = 0.007); however, this interaction became a statistical trend in a sensitivity analysis excluding one outlier in terms of alcohol use. Greater alcohol use and a decrease in frontal pole volume related to longer duration of major depression during follow-up (*p*’s < 0.05). (4) Conclusion: Preliminary findings support more research on alcohol use, PFC trajectories, and depression recurrence in young adults with a mood disorder including individuals with heavier drinking patterns.

## 1. Introduction

Alcohol use/disorders are highly prevalent in mood disorders [1]. Co-occurring mood and alcohol use/disorders are associated with worse clinical outcomes [2,3,4,5,6] characterized by more frequent and severe mood episodes [7,8,9,10], greater cognitive deficits [11,12], increased impulsivity [13], and an elevated risk for suicide [14,15,16,17,18]. Even moderate levels of alcohol consumption are associated with worse clinical outcomes [8]. Despite these data, the mechanisms by which alcohol use alters the illness course are unknown.

Adolescence and young adulthood mark a critical developmental period when mood and alcohol use/disorders typically emerge. The prefrontal cortex (PFC) continues to mature throughout this developmental epoch, with disruptions in typical PFC maturation possibly contributing to the emergence of mood and alcohol use disorders [19,20,21,22,23]. Structural imaging studies show widespread and progressive PFC structural deficits in both mood and alcohol use disorders [24,25,26,27,28,29], possibly serving as a substrate for the high comorbidity of these disorders. Few studies, however, have investigated neural correlates associated with comorbid mood and alcohol use disorders. The few that have converge to suggest PFC structural and functional differences in adults with co-occurring mood and alcohol use disorders, compared to those with a mood disorder alone [30,31,32,33,34].

Prolonged maturation of PFC throughout young adulthood may render the region susceptible to modification by exposure to environmental factors, i.e., alcohol use [35,36,37,38]. Longitudinal neuroimaging studies have shown adolescent alcohol use is associated with accelerated decreases in PFC volume [39,40,41]. An inverse dose-dependent relationship between PFC volume deficits and alcohol use (i.e., quantity, lifetime duration) has also been observed [40,42]. These longitudinal neuroimaging studies focused on typically developing adolescents/young adults, thereby limiting the generalizability of findings to adolescents/young adults with mood disorders. Recent data suggest adolescents/young adults at-risk for and with mood disorders may show distinct neural correlates of alcohol use [43,44]. This could relate to greater sensitivity of the brain to the neurotoxic effects of alcohol. One study of young adults (ages 18–30 years) with bipolar disorder type II and bipolar spectrum disorder observed increased oxidative stress in the anterior cingulate cortex in high-risk drinkers compared to low-risk drinkers [45]. Oxidative stress has been implicated in the pathogenesis of mood disorders and is thought to contribute to illness neuroprogression [46,47]. While results suggest individuals with mood disorders may be susceptible to the neurotoxic effects of alcohol use, the healthy comparison group did not include high-risk drinkers. Additionally, the cross-sectional design limited interpretations of the temporal dynamics between neural trajectories and alcohol use. Indeed, differences in PFC structure are suggested to predate and predict future alcohol use and problems in typically developing young adults and young adults with mood disorders [39,48,49]. Preliminary evidence suggests alcohol use relates to PFC trajectories during adulthood in individuals with major depression or anxiety disorders (average age of 37 years at baseline enrollment [34]). However, longitudinal investigation is needed to disentangle relations among alcohol use, neural progression, and clinical outcomes in mood disorders, particularly during young adulthood when the PFC is still maturing, and symptoms of alcohol misuse often emerge.

The current study used a longitudinal neuroimaging design to examine associations among alcohol use and volumetric trajectories of prefrontal regions of interest (ROIs) in young adults with a mood disorder compared to typically developing young adults. We hypothesized greater alcohol use would be associated with smaller PFC volumes in all young adults, and that young adults with a mood disorder would show a stronger inverse association between baseline alcohol use and a prospective decrease in PFC volume, compared to typically developing young adults. Finally, we also explored relations between alcohol use and alcohol use-related changes in PFC volume with number of weeks with depression and mania over the one-year follow-up period.

## 2. Materials and Methods

### 2.1. Participants

Forty-one young adults (24 with a mood disorder (50% with bipolar disorder and 50% with major depressive disorder)), ages 18–25 years, enrolled in the study and completed one-year follow-up assessments. Participants were recruited through advertising at the University of Texas at Austin campus and in the surrounding area, including local clinics. The majority of participants responded to study advertisements that were recruiting young adults with bipolar disorder, with familial risk for bipolar disorder, and typically developing young adults between 18 and 25 years of age and eligible to complete an MRI scan. Participants completed telephone screening during which they were asked if they had ever been given a mental health diagnosis or seen a therapist/counselor. Following the endorsement of a mental health diagnosis (i.e., unipolar depression or bipolar disorder previously given by a health care provider), participants were asked about depression and mania symptoms of prior mood episodes to screen for those who may not meet criteria for a past major depressive or manic episode according to the Structured Clinical Interview for DSM-5 Research Version (SCID-5-RV) [50]. Participants were also asked if their parents had a diagnosis of bipolar disorder. At enrollment, the SCID-5-RV was used to assess current and lifetime psychiatric diagnoses and clinical characteristics. All participants in the mood disorder group met criteria for a prior mood disorder (major depressive episode or manic episode) according to the SCID-5-RV. The Wechsler Abbreviated Scale of Intelligence-Second Edition (WASI-II) was used as a measure of full-scale intelligence quotient (FSIQ-2) [51]. Current mood symptoms were assessed using the Hamilton Depression Rating Scale (HDRS), Hamilton Anxiety Rating Scale (HARS), and Young Mania Rating Scale (YMRS) [52,53,54]. Exclusion criteria for all participants included IQ < 85, a positive pregnancy test, a history of major medical illness with possible neurological or central nervous system outcomes, or a medical condition or previous surgery preventing participation in magnetic resonance imaging (MRI) scanning. Additional exclusion criteria for typically developing young adults included a history of mood, psychosis, or anxiety disorders, lifetime suicide attempt, or a history of psychotropic medication use. Urinalysis was conducted to assess for substance use and pregnancy on the day of the MRI scan. Participants were asked to abstain from alcohol and drug use 24 h prior to their MRI scan. All participants returned for follow-up assessment, on average 1.2 ± 0.1 years following baseline MRI assessment, to repeat their MRI scan, assessment of recent alcohol use, and the Longitudinal Interval Follow-up Evaluation (LIFE; [55]). The LIFE assessed mood symptoms (depression and mania) over the course of the follow-up period. Table 1 details demographic and clinical characteristics at baseline and follow-up for all participants. All study procedures were approved by and performed in accordance with guidelines and regulations of the University of Texas at Austin Institutional Review Board (IRB approval ID: 2016-10-0114, 7 November 2016). All data were collected prior to March 2020 (before the COVID-19 pandemic-associated mandates and guidelines emerged in the United States). Written informed consent was obtained from all participants in-person before beginning their enrollment study visit.

### 2.2. Structural MRI Acquisition and Preprocessing

All imaging was performed at the University of Texas at Austin Biomedical Imaging Center on a single 3-Tesla Siemens Skyra MR scanner using a 32-channel head coil. Structural MRI images were acquired with a three-dimensional MPRAGE T1-weighted sequence with the following parameters: Repetition time (TR) = 1900 ms, echo time (TE) = 2.42 ms, matrix = 224 × 224, field of view = 220 × 220 mm^2^, 192 one-mm slices without gap and one average. All scans were assessed for movement and noise artifacts. FreeSurfer version 7.1 (https://surfer.nmr.mgh.harvard.edu, accessed on 16 August 2021) was used for cortical surface reconstruction and to obtain measures of volume, as previously described [56]. In brief, automated processing included motion correction [57], removal of non-brain tissue, automated Talairach transformation, segmentation of subcortical white matter and deep volumetric structures, intensity normalization, tessellation of the gray matter/white matter boundary, automated topology correction, and surface deformation following intensity gradients to optimally place the gray/white and gray/cerebrospinal fluid borders. Data pre-processing also included surface inflation, registration to a spherical atlas, parcellation of the cerebral cortex into units with respect to gyrus and sulcus structures, and creation of surface-based data. In FreeSurfer, the Desikan–Killiany automated labeling system was used to parcellate the PFC into gyral-based ROIs (bilateral anterior cingulate cortex (rostral and caudal anterior cingulate cortex combined), orbitofrontal cortex (medial and lateral orbitofrontal cortex combined), and frontal pole) [58]. The anterior cingulate and ventral extending to rostral regions of the PFC were selected based on these regions’ role in the pathophysiology of mood disorders and alcohol use [22,25,41,59]. Table 2 details PFC ROI volumes at baseline and follow-up assessments.

### 2.3. Assessment of Recent Substance Use

Recent alcohol use was measured using a modified version of the Daily Drinking Questionnaire (DDQ) [60] to assess alcohol use during the heaviest drinking week (DDQ-H) over the past month. Total number of drinks during the heaviest drinking week was calculated. Participants were asked at what age they initiated alcohol use (i.e., age of first drink, not just a sip from an adult’s glass, and not including drinking as part of religious ceremonies). The Daily Drug-Taking Questionnaire (DDTQ [61]) was used to assess number of days using cannabis during the heaviest drug-taking week over the past month [62]. Participants completed the DDQ-H and DDTQ again at the follow-up assessment. Table 1 shows alcohol and cannabis use at baseline and follow-up.

### 2.4. Longitudinal Interval Follow-Up Evaluation

Participants completed the Longitudinal Interval Follow-Up Evaluation (LIFE; [55]) at their follow-up visit. This instrument retrospectively collects weekly Psychiatric Status Ratings (PSR) for depression and mania symptoms using a 6-point severity scale (1 = no symptoms, 2–4 = subthreshold symptoms, 5 = meets full threshold DSM criteria for that week, without psychosis or extreme impairment in functioning, 6 = full threshold DSM criteria for that week, with psychosis or extreme impairment in functioning). Number of participants who met full-threshold depression or mania criteria (PSR = 5 or 6) over the follow-up period was calculated. Percentage of weeks participants met the full threshold for depression or mania criteria (PSR = 5 or 6) over the follow-up period was calculated (see Table 3).

### 2.5. Statistical Analyses

#### 2.5.1. Between-Group Differences in Demographics and Baseline Clinical Factors and Alcohol Use

Between-group differences at baseline and follow-up in continuous demographic, clinical, and alcohol/cannabis use variables were assessed with a *t*-test or Wilcoxon test, as appropriate, and included age, IQ, past-week clinical mood symptoms, past-month alcohol (i.e., total number of drinks during heaviest drinking week) and cannabis use (i.e., number of cannabis-use days during heaviest-use week), and percentage of weeks meeting criteria for depression/mania over the follow-up period. Between-group differences in categorical variables were assessed with Chi-square or Fisher’s exact tests as appropriate and included biological sex, number of cannabis users, past/current alcohol or cannabis use disorders, urine toxicology, and presence/absence of a week meeting syndromic criteria for depression and mania over the follow-up period. Within-group changes over time in these factors were also assessed using Wilcoxon Signed Ranked and McNemar’s Chi-Square tests, as appropriate.

#### 2.5.2. Alcohol Use and Prefrontal Cortex Structure

Shapiro–Wilke tests were used to assess normality of the data. A logarithmic transformation was applied to non-normally distributed measures (Shapiro–Wilke test, *p* < 0.05). Main effects of group (mood disorder versus typically developing), time, and baseline alcohol use (total number of drinks during heaviest-drinking week), and interactions among these variables were modeled, with alcohol use and group as the independent variables and PFC ROI volume at baseline and follow-up assessments as the dependent, repeated, within-subject variable (each ROI modeled separately). Models were repeated after removing interaction terms to investigate main effects. Alcohol use at baseline was significantly correlated with alcohol use at follow-up in both groups (typically developing: ρ = 0.9, *p* < 0.0001; mood disorder: ρ = 0.5, *p* = 0.02); therefore, only alcohol use at baseline was included in primary models. Biological sex, time interval between baseline and follow-up MRI assessments, age of alcohol initiation, and total intracranial volume at baseline were included as covariates. Significance was defined as alpha < 0.0083 to account for multiple comparisons (Bonferroni correction for 6 ROI comparisons) for this primary model. Table 4 details model statistics and significant results are reported below. Following a significant group-by-alcohol-use interaction or time-by-group-by-alcohol-use interaction, models were repeated and stratified by group. In order to determine the directionality of findings following a significant time-by-alcohol-use interaction, percent volume change in PFC ROI for each subject was calculated, and associations between baseline alcohol use (independent variable) and percent volume change in PFC ROIs (dependent variable) were investigated, including previous covariates. Additionally, we conducted sensitivity analyses on primary findings controlling for (a) recent cannabis use (number of cannabis-use days during heaviest week in the past 30 days at baseline) and (b) current and past cannabis use disorders at baseline assessment. We also conducted sensitivity analyses controlling for (a) length of time since onset of the first mood episode (calculated as age at baseline minus age of the onset of the first mood episode) and (b) number of prior mood episodes. Finally, we conducted sensitivity analyses excluding outliers (outliers identified as resulting residuals from the main model that lie above/below 1.5 times the interquartile range). A sensitivity analysis excluding any outliers in the alcohol-use independent variable (values that were above/below 1.5 times the interquartile range) was also conducted.

#### 2.5.3. Associations between Alcohol Use and PFC Volume Change with Prospective Mood Symptoms

Associations between alcohol use and prospective duration of depression in the mood disorder group were explored using Spearman correlations. Specifically, alcohol use (total drinks during heaviest-drinking week over the past month) was correlated with percentage of weeks meeting criteria for a depressive episode (PSR = 5–6) during the follow-up period. Associations were also explored between change in PFC volume (only ROIs that showed a significant relation with alcohol use in the primary models) and prospective depression in the mood disorder group. Specifically, percent volume change in each PFC ROI showing a time-by-alcohol-use interaction was calculated for each participant with a mood disorder. Spearman correlations were used to assess relations between percent volume change in PFC ROIs with percentage of weeks meeting criteria for a depressive episode during the follow-up period. These models were repeated (only including those with bipolar disorder) to investigate relations with percentage of weeks meeting criteria for a manic episode during the follow-up period. One participant converted from major depressive disorder to bipolar disorder over the follow-up period and was included in the models investigating mania. Significance was defined as alpha < 0.05 for these exploratory analyses. Following a significant relation between PFC ROI volume and prospective mood symptoms, we further explored relations between prospective depressive symptoms and changes in brain volume by repeating primary models (as described in Methods Section 2.5.2) with prospective mood symptoms replacing alcohol use as the independent variable.

#### 2.5.4. Exploratory Analysis on the Effect of Mood Disorder Diagnosis

Primary models, as described above, were repeated within the mood disorder group to explore interactions with diagnosis (bipolar disorder or major depressive disorder). Specifically, main effect of diagnostic group (bipolar disorder versus major depressive disorder), time, and alcohol use at baseline, and interactions among these variables were modeled. Alcohol use as the independent variable and PFC ROI volume at baseline and follow-up assessments were the dependent, repeated, within-subject variables (each ROI modeled separately), including covariates described above. The participant who converted from major depressive disorder to bipolar disorder over the follow-up period was included in the bipolar disorder group for analyses.

## 3. Results

### 3.1. Between Group Differences in Demographics and Baseline Clinical Factors

The mood disorder group had more women than the typically developing group. Compared to the typically developing group, the mood disorder group also exhibited greater depression and anxiety symptoms at baseline and greater depression, anxiety, and mania scores at follow-up. No other between-group differences or within-group changes over time (baseline to follow-up) were observed. There were no significant between-group differences in alcohol use at baseline or follow-up, and groups did not show a significant change in alcohol use over the one-year follow-up.

### 3.2. Alcohol Use and Prefrontal Cortex Structure

**Figure 1:**
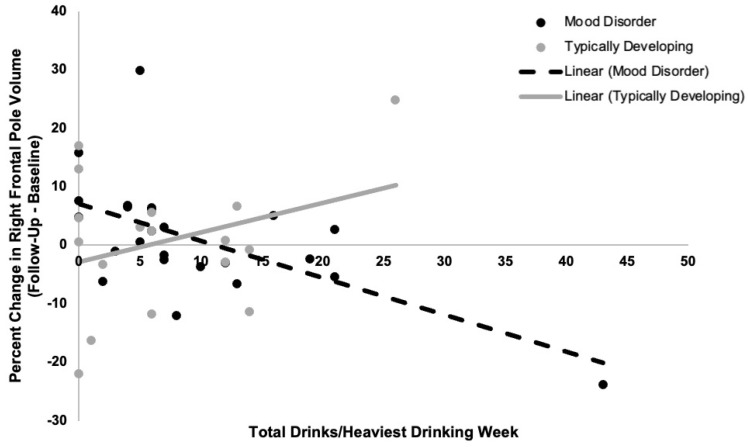
Relations between recent quantity of alcohol use (total drinks per week) and percent change in right frontal pole volume from baseline to follow-up in typically developing young adults and young adults with a mood disorder. A time-by-group-by-alcohol-use interaction on right frontal pole volume was observed (*p* = 0.007). Greater recent alcohol use related to decreases in right frontal pole volume from baseline to follow-up assessments in young adults with a mood disorder (r^2^ = 0.6, *p* = 0.0005). There was no significant relationship between recent alcohol use and change in right frontal pole volume from baseline to follow-up in typically developing young adults (*p* = 0.3).

We observed a time-by-group-by-alcohol-use interaction in right frontal pole volume (F_(1,33)_ = 8.3, *p* = 0.007). Stratifying by group revealed a significant time-by-alcohol-use interaction on right frontal pole volume in young adults with a mood disorder (F_(1,18)_ = 23.2, *p* = 0.0001) but not in typically developing participants (F_(1,11)_ = 1.3, *p* = 0.3). Specifically, greater alcohol use was associated with a greater decrease in percent volume in right frontal pole in young adults with a mood disorder (t = −4.2, *p* = 0.0005; see Figure 1). Results remained significant when controlling for (a) recent cannabis use and (b) current and past cannabis use disorders, and when controlling for (a) length of time since the onset of first mood episode and (b) number of prior mood episodes. Results also remained significant when excluding two participants (one with a mood disorder and one typically developing) who were identified as outliers. However, when excluding a participant (in the mood disorder group) who was identified as an outlier in terms of alcohol use, the observed time-by-group-by-alcohol-use interaction became a trend (F_(1,32)_ = 3.3, *p* = 0.08). When stratifying by group, the time-by-alcohol-use interaction on right frontal pole volume in young adults with a mood disorder remained significant (F_(1,17)_ = 4.3, *p* = 0.05) and the model in the typically developing group remained nonsignificant.

### 3.3. Associations between Alcohol Use and Volume Change with Prospective Mood Symptoms

Greater quantity of alcohol use at baseline was prospectively associated with greater percentage of weeks with syndromic depression (ρ = 0.5, *p* = 0.01). Greater percent decrease in right frontal pole volume over the follow-up period was associated with greater percentage of weeks with syndromic depression (ρ = −0.5, *p* = 0.03). There were no significant interactions with, or effects of, prospective depression symptoms on right frontal pole volume when repeating primary models and replacing alcohol with prospective depression symptoms (time-by-group-by-prospective-depression-symptoms interaction on right frontal pole volume, F_(1,33)_ = 0.5, *p* = 0.5; group-by-prospective-depression-symptoms interaction, F_(1,33)_ = 0.3, *p* = 0.6; main effect of prospective depression symptoms, F_(1,34)_ = 0.4, *p* = 0.5).

### 3.4. Exploratory Analysis on the Effects of Mood Disorder Diagnosis

There was no main effect of, or interaction with, mood disorder diagnosis on PFC volume.

## 4. Discussion

Results may support our hypothesis that greater alcohol use in young adults (on average, at 21 years of age) is prospectively associated with decreased frontal pole volume over time in those with a mood disorder. We did not observe an association between baseline alcohol use and frontal pole structural trajectory in the typically developing group. While, on average, the current sample exhibited low to moderate levels of alcohol use, one participant in the mood disorder group was identified as an outlier on the alcohol use measure, i.e., exhibited heavier alcohol use compared to others. When removing this participant, the observed time-by-group-by-alcohol-use interaction became a statistical trend, while the time-by-alcohol-use interaction within the mood disorder group remained significant. This negative finding could support no relation between alcohol use and PFC trajectories; however, it could also suggest group differences in relations between alcohol use and brain structure may be more subtle when confining analyses to an examination of low to moderate levels of alcohol consumption.

The current study also found alcohol use and associated decreases in frontal pole volume related to prospective clinical trajectories. Specifically, greater alcohol use at baseline and prospective decrease in frontal pole volume was associated with a greater percentage of weeks meeting criteria for major depression over the follow-up period in young adults with a mood disorder. This is in line with prior work suggesting low to moderate levels of alcohol use are associated with worse clinical outcomes in mood disorders [8]. However, larger samples, including those with heavier drinking patterns, are needed to test these relations across a range of consumption levels.

Alcohol use, even in low to moderate levels [8], adversely influences the clinical course of mood disorders, and results from the current study suggest this may be related to differences in the structural trajectory of the frontal pole. Individuals with mood disorders have shown volumetric deficits localized to the frontal pole [24,26,63], with frontal pole volumetric measures inversely associated with illness severity and duration [63,64]. The frontal pole plays an important integratory role in higher-order emotional and cognitive processes [65], including decision making and cognitive inhibition [66,67]. Structural abnormalities in this region have been suggested to contribute to behavioral disturbances commonly observed in depression (i.e., introspective evaluation [68], self-reflection [69], and rumination [70]). While speculative, it is possible alcohol use contributed to structural changes in the frontal pole in young adults with a mood disorder. Chitty and colleagues (2013) found alcohol use exacerbates PFC oxidative stress—a mechanism thought to contribute to disease neuroprogression—in individuals (ages 18–30) with bipolar disorder type II and bipolar spectrum disorder [45]. Taken together, results suggest this region of the PFC may be more sensitive to the neurotoxic effects of alcohol use in young adults with a mood disorder relative to typically developing young adults. Studies have also shown greater PFC volume decreases over time in adolescents with bipolar disorder relative to typically developing controls [27], and it is also possible that differences in PFC developmental trajectories—present prior to baseline MRI assessment—contributed to greater alcohol use. Indeed, smaller volume in the PFC, including in the area of the frontal pole, has been observed in youth with bipolar disorder and suggested to distinguish those that prospectively initiate and develop alcohol use problems [49]. These interpretations are not mutually exclusive; alcohol use may interact with neural vulnerability in youth with a mood disorder to increase the risk of developing alcohol use disorders over time.

There was a relation between alcohol use and brain volume in the frontal pole but not in the orbitofrontal or anterior cingulate cortices. Brain maturation occurs in a posterior to anterior progression. While we can only speculate, it is possible that developmental differences in the frontal pole may render it particularly susceptible to low/moderate levels of alcohol use during the young adult period. The orbitofrontal and anterior cingulate cortical ROIs used in this study were larger in size relative to the frontal pole ROI. It is also possible that the larger ROI size decreased sensitivity to detect a relation between alcohol use and orbitofrontal/anterior cingulate cortical brain structure.

The typically developing group did not show a significant association between alcohol use and PFC volume trajectories. This deviates from prior studies reporting adolescent alcohol use are associated with accelerated decreases in PFC volume in typical development [39,40]. Discrepant findings may stem from differences in the amount of alcohol use reported between study samples. The current study sample was comprised of low to moderate drinkers, while prior studies included participants reporting heavier recent alcohol use. It is also possible inconsistent findings are related to differences in the age ranges of study participants. Prior studies have focused on adolescents/young adults between the ages of 12 and 21, with an average age between 13 and 16 years old at enrollment [39,40,41], whereas the current study focused on the older age range of 18 to 25, with an average age of 21 years at enrollment. It is possible that in typical development, the PFC is more susceptible to alcohol-related insults during a younger adolescent age period, or differences may “normalize” over time in youth that do not develop alcohol use disorders. In line with prior work [38,71], we found greater baseline alcohol use was associated with lower right frontal pole volume across all young adults, although this result did not survive correction for multiple comparisons. The PFC continues to develop into the mid-20′s [72], and larger longitudinal studies including heavier drinking samples and multiple follow-up assessments are needed to clarify the likely bidirectional relationship between alcohol and PFC structural trajectories, as well as interactions with other factors that can mitigate these relations (e.g., physical exercise [73]).

### Limitations

Several limitations must be considered when interpreting these findings. Sample size was small, limiting power for all but primary contrasts. The mixed mood disorder group included young adults with bipolar disorder and major depressive disorder, introducing heterogeneity that might have further weakened statistical power. Structural brain differences have been observed between bipolar disorder and major depressive disorder [28]. While underpowered, we did not observe main effects or interactions of mood disorder diagnosis on PFC findings. During phone screening, 83% of participants with unipolar depression reported having a parent with bipolar disorder, 8% of participants with bipolar disorder reported having a parent with bipolar disorder, and 18% of the typically developing comparison group reported having a parent with bipolar disorder. While this study did not directly assess diagnosis in parents of the participants enrolled in this study, and hence cannot confirm a parental diagnosis of bipolar disorder, this finding does suggest the depression group may be a more homogenous group associated with familial risk for bipolar disorder. Future studies, with larger and more homogeneous samples, are needed to replicate and extend these findings, including investigating familial risk factors that may contribute to these outcomes. Additionally, the majority of the mood disorder group was female. Sex differences in the neural correlates of alcohol use have been documented [49,74], and while our models included biological sex as a covariate, we were underpowered to investigate sex differences. A subset of participants in each group reported recent cannabis use. While cannabis use could have influenced measures of PFC volume, results were significant after controlling for recent cannabis use and past/current cannabis use disorders. We did not evaluate medication use over the entire follow-up period and were underpowered to investigate the effects of medications in the mood disorder group. Psychotropic medication use has been shown to affect brain structure [75] and may also interact with alcohol use to impact neural and clinical outcomes. Higher-powered studies should control for and explore interactions with these factors, including sex, recent drug use, and medication. However, the heterogeneous nature of our mood disorder group increases the generalizability of findings. The study relied on a retrospective self-report measure to assess recent alcohol use and the possibility of inaccurate recall cannot be excluded. The LIFE was designed to evaluate mood symptoms over a 6-month period. The current study utilized this instrument to evaluate depression and mania symptoms over a one-year follow-up period. Especially considering this longer evaluation period, the possibility of inaccurate recall must also be considered for this retrospective self-report measure. The current study cannot determine whether alcohol use contributed to frontal pole volume changes or if alcohol use emerged in part due to differences in PFC developmental trajectories present prior to baseline MRI assessment. Additionally, depression symptom recurrence may be more directly associated with greater loss of PFC volume than greater alcohol use per se, and individuals who are more depressed may drink to self-medicate the depressive symptoms. These processes are undoubtedly complex, and brain changes likely stem from multiple interacting factors. Prospective investigation beginning prior to alcohol use initiation and with more frequent assessment of alcohol use and mood symptoms patterns is needed to better understand the temporal dynamics between these factors. Alcohol use has a detrimental impact on the clinical course of mood disorders; preliminary findings support the need to confirm and extend these findings and investigate how alcohol use—ranging from light to heavy consumption—relates to neural and clinical progression in mood disorders to better inform clinical recommendations for patients and mitigate the adverse consequences of alcohol use in young adults with mood disorders.

## Figures and Tables

**Table 1:** Demographic and clinical characteristics in typically developing young adults and young adults with a mood disorder at baseline and follow-up assessments.

		Baseline			Follow-Up			Within Group Changes over Time	
		Typically Developing (N = 17)	Mood Disorder (N = 24)	*p*-Value	Typically Developing (N = 17)	Mood Disorder (N = 24)	*p*-Value	Typically Developing *p*-Value	Mood Disorder *p*-Value
Demographics	Mean Age (SD)	20.5 (1.4)	20.8 (2.0)	0.6	21.7 (1.4)	22.0 (1.9)	0.6	---	---
	Number of Females (%)	9 (53)	20 (83)	*0.05 ^F^*	---	---	---	---	---
	Mean WASI-II FSIQ ^A^	115.8 (12.5)	118.8 (11.7)	0.4	---	---	---	---	---
Clinical Mood Symptoms	HDRS ^B^ (SD)	2.5 (3.1)	7.8 (6.3)	*0.002 ^Z^*	2.8 (3.9)	8.0 (6.5)	*0.002 ^Z^*	0.9 ^S^	0.5 ^S^
	HARS ^C^ (SD)	2.4 (3.3)	7.4 (7.0)	*0.002 ^Z^*	3.2 (3.7)	7.8 (7.1)	*0.02 ^Z^*	0.4 ^S^	0.6 ^S^
	YMRS ^D^ (SD)	0.6 (1.5)	1.4 (3.4)	0.9 ^Z^	0.1 (0.2)	1.4 (2.4)	*0.005 ^Z^*	0.06 ^S^	0.8 ^S^
Mood Disorder	Major Depressive Disorder (%)	---	12 (50)	---	2 (12)	11 (46)	---	0.5 *^M^*	1 ^M^
	Bipolar Disorder (%)	---	12 (50)	---	---	13 (54)	---	---	1 ^M^
Alcohol/ Cannabis Use Disorders (A/CUD)	Current AUD, mild (%)	0	0	1 ^F^	1 (6)	0	0.4 ^F^	1 ^M^	---
	Current AUD, moderate (%)	0	0	1 ^M^	1 (6)	2 (8)	1 ^F^	1 ^M^	0.5 ^M^
	Past AUD, mild (%)	0	1 (4)	1 ^F^	0	1 (4)	1 ^F^	1 ^M^	1 ^M^
	Current CUD, mild (%)	0	1 (4)	1 ^F^	0	3 (13)	0.3 ^F^	1 ^M^	0.6 ^M^
	Current CUD, moderate (%)	1 (6)	1 (4)	1 ^F^	0	2 (8)	1 ^F^	1 ^M^	1 ^M^
	Current CUD, severe (%)	0	1 (4)	1 ^F^	1 (6)	0	0.4 ^F^	1 ^M^	1 ^M^
	Past CUD, mild (%)	0	2 (8)	0.5 ^F^	1 (6)	2 (8)	1 ^F^	1 ^M^	0.5 ^M^
Recent Alcohol and Cannabis Use	Total Drinks/Week ^E^ (SD)	6.9 (7.3)	9.4 (9.5)	0.3 ^Z^	8.9 (9.0)	8.7 (8.9)	0.9 ^Z^	0.08 ^S^	0.08 ^S^
	Cannabis Users (%)	7 (41)	10 (42)	1.0	7 (41)	11 (46)	0.8	0.7 ^S^	1 ^S^
	Cannabis Use Days/Week ^G^ (SD)	1.7 (2.7)	1.7 (2.5)	1.0 ^Z^	1.1 (1.9)	1.7 (2.6)	0.6 ^Z^	0.5 ^S^	0.6 ^S^
Positive Urinalysis Toxicology Screen	Tetrahydrocannabinol (%)	2 (12)	7 (29)	0.3 ^F^	2 (12)	7 (29)	0.3 ^F^	0.5 ^M^	0.6 ^M^
	Cocaine (%)	0	1 (4)	1 ^F^	0	1 (4)	1 ^F^	1 ^M^	1 ^M^
	Amphetamines (%)	0	2 (8)	0.5 ^F^	0	3 (13)	0.3 ^F^	1 ^M^	1 ^M^
	Benzodiazepines (%)	0	1 (4)	1 ^F^	0	1 (4)	1 ^F^	1 ^M^	0.5 ^M^
	Phencyclidines (%)	0	1 (4)	1 ^F^	0	1 (4)	1 ^F^	1 ^M^	0.5 ^M^
Clinical Factors & Comorbidities	Lifetime Suicide Attempt (%)	---	6 (25)	---	---	6 (25)	---	---	1 ^M^
	Comorbid Anxiety Disorders ^H^ (%)	---	5 (21)	---	---	6 (25)	---	---	1 ^M^
Medications ^I^	Unmedicated at scan (%)	17	14 (58)	---	15	15 (63)	---	0.5 ^M^	1 ^M^
	Antispychotic (%)	0	4 (17)	---	0	4 (17)	---	1 ^M^	1 ^M^
	Anticonvulsant (%)	0	3 (13)	---	0	4 (17)	---	1 ^M^	1 ^M^
	Antidepressant/SSRIs (%)	0	2 (8)	---	2 (12)	3 (13)	1 ^F^	0.5 ^M^	1 ^M^
	Stimulant (%)	0	2 (8)	---	0	2 (8)	---	1 ^M^	0.5 ^M^
	Lithium (%)	0	4 (17)	---	0	4 (17)	---	1 ^M^	0.5 ^M^
	Anxiolytics (%)	0	1 (4)	---	0	0	---	1 ^M^	1 ^M^
	Sedatives/Antihistamines (%)	0	2 (8)	---	0	0	---	1 ^M^	0.5 ^M^

Between-group differences in age and FSIQ-2 at baseline were compared using a two-sample *t*-test. All other factors were examined with a Mann–Whitney–Wilcoxon or Fisher Exact test, as appropriate. Between-group differences in lifetime suicide attempt, lifetime anxiety disorders, and psychotropic medications at baseline were not assessed because these factors were considered an exclusion criterion, and thus, not present in the typically developing group. Wilcoxon Signed Ranked tests were used to assess changes in clinical mood symptoms and alcohol/cannabis use over time (baseline to follow-up). McNemar’s Chi-Square Tests were used to assess changes over time in all other factors. ^F^ represents *p*-value calculated with Fisher exact test. ^Z^ represents *p*-value calculated with a Mann–Whitney–Wilcoxon Test. ^S^ represents *p*-value calculated with a Wilcoxon Signed Ranked test. ^M^ represents *p*-value calculated with a McNemar Chi-Square Test. ^A^ FSIQ-2 represents the composite score for the full-scale intelligence quotient comprising verbal comprehension and matrix reasoning subtests on the Wechsler Abbreviated Scale of Intelligence Second Edition (WASI-II). ^B^ Past week depression symptoms were measured using the Hamilton Depression Rating Scale (HDRS). ^C^ Past week anxiety symptoms were measured using the Hamilton Anxiety Rating Scale (HARS). ^D^ Past week mania symptoms were measured using the Young Mania Rating Scale (YMRS). ^E^ Recent alcohol use was measured with the Daily Drinking Questionnaire adapted for heaviest week over the past month (DDQ-H). ^G^ Recent cannabis use at baseline assessment was measured with the Daily Drug-Taking Questionnaire adapted for heaviest drug-taking week over the past month (DDTQ). ^H^ Comorbid anxiety disorders included generalized anxiety disorder and panic disorder. ^I^ Medication use was assessed at time of MRI evaluation and was required to be stable over past 30 days.

**Table 2:** Region of interest volumes (mm^3^) at baseline and follow-up.

	Baseline		Follow-Up	
	Typically Developing (N = 17)	Mood Disorder (N = 24)	Typically Developing (N = 17)	Mood Disorder (N = 24)
Left Orbitofrontal Cortex Volume (SD)	14,601 (1810)	14,079 (1290)	14,524 (1753)	14,106 (1394)
Right Orbitofrontal Cortex Volume (SD)	14,785 (1781)	14,416 (1102)	14,788 (1771)	14,287 (1248)
Left Anterior Cingulate Cortex Volume (SD)	4942 (920)	4888 (928)	4905 (840)	4873 (839)
Right Anterior Cingulate Cortex Volume (SD)	4537 (751)	4497 (770)	4541 (864)	4637 (1030)
Left Frontal Pole Volume (SD)	1318 (197)	1162 (198)	1281 (174)	1215 (186)
Right Frontal Pole Volume (SD)	1599 (298)	1519 (183)	1588 (210)	1532 (206)

Region of interest (ROI) volumes in typically developing young adults and young adults with a mood disorder at baseline and follow-up assessments.

**Table 3:** Psychiatric Status Ratings for depression and mania over the follow-up period.

	Typically Developing (N = 17)	Mood Disorder (N = 24)	*p*-Value
Met Criteria for Major Depressive Episode (%)	2 (12)	9 (38)	0.09 ^F^
Average Duration: % Weeks met Criteria for Major Depressive Episode (SD); range	1 (2); 0–8	12 (21); 0–77	0.06 ^Z^
Met Criteria for Mania (%) ^A^	---	5 (21)	---
Average Duration: % Weeks met Criteria for Mania (SD); range ^A^	---	7 (17); 0–73	---

The Longitudinal Interval Follow-up Evaluation was used to assess Psychiatric Status Ratings (PSR) for depression and mania using a 6-point severity scale (1 = no symptoms, 2–4 = subthreshold symptoms, 5 = meets full threshold DSM-4 criteria for that week, without psychosis or extreme impairment in functioning, 6 = full threshold DSM-4 criteria for that week, with psychosis or extreme impairment in functioning). Percentage (%) of weeks participants met full-threshold DSM-4 criteria for depression or mania (PSR = 5 or 6) over the follow-up period was also calculated. Between-group (mood disorder versus typically developing) differences in number of participants meeting full-threshold criteria for depression/mania symptoms were calculated using Chi-square or Fisher’s exact tests, as appropriate. Between-group differences in duration of depression/mania symptoms were calculated with a Mann–Whitney–Wilcoxon test. ^A^ Average percentage of participants meeting criteria for mania and average duration of mania over the follow-up period was only assessed in participants with bipolar disorder. ^F^ represents *p*-value calculated with Fisher exact test. ^Z^ represents *p*-value calculated with a Mann–Whitney–Wilcoxon Test.

**Table 4:** Model statistics for prefrontal cortex regions of interest.

	Orbitofrontal Cortex		Anterior Cingulate Cortex		Frontal Pole	
	Left	Right	Left	Right	Left	Right
Time	F_(1,34)_ = 6.7, *p* = 0.01	F_(1,34)_ = 4.9, *p* = 0.03	F_(1,34)_ = 2.5, *p* = 0.1	F_(1,34)_ = 3.4, *p* = 0.08	F_(1,34)_ = 0.3, *p* = 0.6	F_(1,34)_ = 0.01, *p* = 0.9
Group	F_(1,34)_ = 0.4, *p* = 0.5	F_(1,34)_ = 0.4, *p* = 0.5	F_(1,34)_ = 0.4, *p* = 0.5	F_(1,34)_ = 0.03, *p* = 0.9	F_(1,34)_ = 1.5, *p* = 0.2	F_(1,34)_ = 0.02, *p* = 0.9
Alcohol Use	F_(1,34)_ = 0.2, *p* = 0.7	F_(1,34)_ = 0.005, *p* = 0.9	F_(1,34)_ = 0.1, *p* = 0.7	F_(1,34)_ = 1.7, *p* = 0.2	F_(1,34)_ = 2.6, *p* = 0.1	F_(1,34)_ = 5.1, *p* = 0.03
Time × Group	F_(1,34)_ = 0.03, *p* = 0.9	F_(1,34)_ = 0.3, *p* = 0.6	F_(1,34)_ = 0.03, *p* = 0.9	F_(1,34)_ = 0.7, *p* = 0.4	F_(1,34)_ = 3.4, *p* = 0.07	F_(1,34)_ = 0.01, *p* = 0.9
Time × Alcohol Use	F_(1,34)_ = 0.0004, *p* = 1.0	F_(1,34)_ = 1.0, *p* = 0.3	F_(1,34)_ = 0.02, *p* = 0.9	F_(1,34)_ = 0.08, *p* = 0.8	F_(1,34)_ = 1.4, *p* = 0.2	F_(1,34)_ = 3.3, *p* = 0.08
Group × Alcohol Use	F_(1,33)_ = 0.02, *p* = 0.9	F_(1,33)_ = 0.9, *p* = 0.3	F_(1,33)_ = 0.08, *p* = 0.8	F_(1,33)_ = 0.05, *p* = 0.8	F_(1,33)_ = 2.5, *p* = 0.1	F_(1,33)_ = 3.6, *p* = 0.07
Time × Group × Alcohol Use	F_(1,33)_ = 1.0, *p* = 0.3	F_(1,33)_ = 4.0, *p* = 0.05	F_(1,33)_ = 0.03, *p* = 0.9	F_(1,33)_ = 0.001, *p* = 1.0	F_(1,33)_ = 0.03, *p* = 0.9	F_(1,33)_ = 8.3, *p = 0.007*

Results are reported for main effects of time, group, and alcohol use, as well as time-by-group, time-by-alcohol-use, group-by-alcohol-use, and time-by-group-by-alcohol-use interactions. Models were repeated after removing interaction terms to investigate main effects. Significance was defined as alpha <0.0083 to account for multiple comparisons (Bonferroni correction for 6 ROI comparisons).

## Data Availability

The data that support the findings of this study are available from the corresponding author upon reasonable request.
